# Supplementary material for: The prevalence of overweight and obesity and the assessment of associated risk factors among school-aged adolescents in Kandahar City, Afghanistan
Source: PLoS One. 2025 Oct 27;20(10):e0335359. doi: 10.1371/journal.pone.0335359 (PMC12558500; doi:10.1371/journal.pone.0335359)
Supplement: Attachment — Submitted filename: Comments_PONE_D_25_33318.docx [file pone.0335359.s002.docx]

**Reviewer Comments**

**General Assessment**

This manuscript addresses a relevant public health concern: the prevalence and risk factors of adolescent overweight and obesity in Kandahar, Afghanistan. The topic is timely, and the study provides much-needed local data that could inform interventions and policies. The study design, data collection, and analysis are generally appropriate. However, there are several methodological limitations, presentation issues, and clarity concerns that should be addressed before publication.

**Major Comments**

1. **Sample Representation**

The study exclusively included male adolescents, which limits generalizability. While cultural and political restrictions are understandable, this limitation should be emphasized more prominently in the Abstract, Methods, Discussion, and Limitations sections. Consider rephrasing the conclusion to reflect that results apply to male adolescents only.

2. **BMI Classification Standards**

The manuscript uses CDC BMI percentiles. Please justify why CDC references were chosen over WHO growth standards, which are more globally accepted for international comparisons.

3. **Abstract and Key Findings**

The abstract should briefly mention the main limitation (male-only sample) to provide a balanced overview.

“Screen time ≥30 minutes/day” seems like a very low threshold for obesity risk. Please clarify whether this cutoff reflects your data distribution, or if a higher standard threshold (e.g., ≥2 hours/day) was not available. This requires explanation in the Methods and Discussion.

4. **Results Presentation**
a) Some tables are too detailed (e.g., socio-demographics by 15 districts). Consider simplifying or moving such information to supplementary material.
b) There is a formatting error in the Results: “Error! Reference source not found.” — this must be corrected.
c) Clarify why certain variables (e.g., cold drink consumption, physical activity) that were close to significance in bivariate analysis were excluded from the final model.

**Discussion**

While comprehensive, the Discussion is too long and sometimes reads like a review article (e.g., genetic pathways, detailed comparisons with other countries). Consider shortening to focus on:

a) The key findings of this study.
b) How they compare with previous Afghan and regional studies.
c) What is novel about your data.
d) Clarify the distinction between this study and the earlier Rahimi et al. (2025) Kandahar study. Both are cited, but it is not clear how they differ.

**Strengths and Limitations**

Limitations should be more explicit and upfront:

a) Male-only sample.
b) Self-reported lifestyle data (recall and social desirability bias).
c) Urban-only setting, limiting generalizability to rural Afghanistan.
d) Cross-sectional design precludes causal inference.

**Recommendations**

Currently, recommendations are broad. Strengthen them by tying directly to findings. For example:

a) Since school canteen food was a significant risk factor, suggest policy interventions for healthier school canteen menus.
b) Since screen time was significant, recommend structured school-based physical activity programs to counter sedentary behavior.

**Minor Comments**

**Introduction**

Streamline repetitive statistics (global obesity prevalence is presented in multiple ways).

Avoid futuristic references (e.g., “WHO 2025” — please check citation year accuracy).

Line 69 you mentioned prevalence has risen…….. indicate statistics to give wait ie from what percentage to what??

Line 96 “therefore the objective of….”, fills irrelevant. Just state, therefore, this study aimed to……….

**Methods**

a) Line 104 through 107: the information here is not required since it has details in the subsequent sections. Please consider only talking about the design.
b) Line 117, I suggest the authors give the justification for not including females in the study or why the males were considered.
c) Anthropometric measurement description is overly detailed; could be shortened without losing rigor.

**Tables**

a) Ensure consistency in formatting (e.g., percentages with one decimal place).
b) Combine or summarize where possible for clarity.

**References**

a) There are duplicates (e.g., Rahimi et al., 2025 appears twice). Please check and merge.
b) Some citations (e.g., [7], WHO fact sheet) have unusual formatting and need standardization.

**Language and Style**

a) Minor grammatical issues (e.g., “were not engage” → “were not engaged”).
b) Improve flow in the objectives paragraph (“assess the prevalence… investigate… provide evidence-based recommendations” could be restructured into a single clear sentence).

I recommend major revisions before this manuscript can be considered for publication. The study has merit and provides important local evidence, but improvements are needed in methodology reporting, clarity, and presentation.
