# Supplementary material for: The prevalence of overweight and obesity and the assessment of associated risk factors among school-aged adolescents in Kandahar City, Afghanistan
Source: PLoS One. 2025 Oct 27;20(10):e0335359. doi: 10.1371/journal.pone.0335359 (PMC12558500; doi:10.1371/journal.pone.0335359)
Supplement: Attachment — Submitted filename: Response to Reviewers...docx [file pone.0335359.s003.docx]

**Reviewer Comments**

**General Assessment**

This manuscript addresses a relevant public health concern: the prevalence and risk factors of adolescent overweight and obesity in Kandahar, Afghanistan. The topic is timely, and the study provides much-needed local data that could inform interventions and policies. The study design, data collection, and analysis are generally appropriate. However, there are several methodological limitations, presentation issues, and clarity concerns that should be addressed before publication.

**Major Comments**

1. **Sample Representation**

The study exclusively included male adolescents, which limits generalizability. While cultural and political restrictions are understandable, this limitation should be emphasized more prominently in the Abstract, Methods, Discussion, and Limitations sections. Consider rephrasing the conclusion to reflect that results apply to male adolescents only.

**Answer**: Thanks. The main reason for male predominance in this study was due to restrictions on female education. Now this is mentioned in the Abstract, Methods, Discussion, and Limitations sections.

2. **BMI Classification Standards**

The manuscript uses CDC BMI percentiles. Please justify why CDC references were chosen over WHO growth standards, which are more globally accepted for international comparisons.

**Answer**: Yes, we totally agree with you. The use of CDC instead of WHO standards was the comment of the Kandahar University Ethics Committee. The committee said that we should use CDC BMI percentiles. So, we had no choice but to accept their comments.

3. **Abstract and Key Findings**

The abstract should briefly mention the main limitation (male-only sample) to provide a balanced overview.

“Screen time ≥30 minutes/day” seems like a very low threshold for obesity risk. Please clarify whether this cutoff reflects your data distribution, or if a higher standard threshold (e.g., ≥2 hours/day) was not available. This requires explanation in the Methods and Discussion.

**Answer**: Thanks. Yes, you are right. For analytical purposes, we categorized screen time using a cutoff of ≥30 minutes/day. This threshold was based on the data distribution in our sample, as a majority of participants reported very low screen exposure. While international guidelines often use ≥2 hours/day as the risk threshold, this higher cutoff was not practical for analysis in our dataset due to the small number of adolescents exceeding that level.

4. **Results Presentation**
a) Some tables are too detailed (e.g., socio-demographics by 15 districts). Consider simplifying or moving such information to supplementary material.

**Answer**: Thanks. Now the tables have been shortened.

b) There is a formatting error in the Results: “Error! Reference source not found.” — this must be corrected.

**Answer**: Thanks. Now the error is corrected.

c) Clarify why certain variables (e.g., cold drink consumption, physical activity) that were close to significance in bivariate analysis were excluded from the final model.

**Answer**: Thanks. We used the acceptable statistically significant value of <0.05. For cold drink consumption, it was 0.06, while 0.4 for physical activity. Both of them were >0.05, so we excluded them.

**Discussion**

While comprehensive, the Discussion is too long and sometimes reads like a review article (e.g., genetic pathways, detailed comparisons with other countries). Consider shortening to focus on:

a) The key findings of this study.
b) How they compare with previous Afghan and regional studies.
c) What is novel about your data.

**Answer**: Thanks for the nice comment. Now we have shortened it.

d) Clarify the distinction between this study and the earlier Rahimi et al. (2025) Kandahar study. Both are cited, but it is not clear how they differ.

**Answer**: Thanks. Yes, it was a mistake. Both are the same study. Now corrected.

**Strengths and Limitations**

Limitations should be more explicit and upfront:

a) Male-only sample.
b) Self-reported lifestyle data (recall and social desirability bias).
c) Urban-only setting, limiting generalizability to rural Afghanistan.
d) Cross-sectional design precludes causal inference.

**Answer**: Thanks. Now limitations have been made more explicit and upfront.

**Recommendations**

Currently, recommendations are broad. Strengthen them by tying directly to findings. For example:

a) Since school canteen food was a significant risk factor, suggest policy interventions for healthier school canteen menus.
b) Since screen time was significant, recommend structured school-based physical activity programs to counter sedentary behavior.

**Answer**: Thanks. Now recommendations have been reviewed and changes made accordingly.

**Minor Comments**

**Introduction**

Streamline repetitive statistics (global obesity prevalence is presented in multiple ways).

Avoid futuristic references (e.g., “WHO 2025” — please check citation year accuracy).

Line 69 you mentioned prevalence has risen…….. indicate statistics to give wait ie from what percentage to what??

Line 96 “therefore the objective of….”, fills irrelevant. Just state, therefore, this study aimed to……….

**Answer**: Thanks. Now we have made the changes in the manuscript accordingly.

**Methods**

a) Line 104 through 107: the information here is not required since it has details in the subsequent sections. Please consider only talking about the design.

**Answer**: Thanks. Now the information is removed.

b) Line 117, I suggest the authors give the justification for not including females in the study or why the males were considered.

**Answer**: Thanks. It was due to ban on female education in Afghanistan. We have mentioned this issue in the abstract, methods, discussion, and conclusion sections.

c) Anthropometric measurement description is overly detailed; could be shortened without losing rigor.

**Answer**: Thanks. Now anthropometric measurement description is shortened.

**Tables**

a) Ensure consistency in formatting (e.g., percentages with one decimal place).

**Answer**: Thanks. OK, now consistency has been brought in describing the data.

b) **Combine or** summarize where possible for clarity.

**Answer**: Thanks. Now tables have been summarized.

**References**

a) There are duplicates (e.g., Rahimi et al., 2025 appears twice). Please check and merge.

**Answer**: Thanks. This was a mistake. Now corrected.

b) **Some citations (**e.g., [7], WHO fact sheet) have unusual formatting and need standardization.

**Answer**: Thanks. Now they have been standardized.

**Language and Style**

a) Minor grammatical issues (e.g., “were not engage” → “were not engaged”).

**Answer**: Thanks. Now the whole manuscript is thoroughly checked and grammatical issues solved.

b) Improve flow in the objectives paragraph (“assess the prevalence… investigate… provide evidence-based recommendations” could be restructured into a single clear sentence).

**Answer**: Thanks. Now this problem is solved.

I recommend major revisions before this manuscript can be considered for publication. The study has merit and provides important local evidence, but improvements are needed in methodology reporting, clarity, and presentation.

Reviewer #1: Thank you for the opportunity to read this manuscript! I think it will be a good value add to the literature. I have a few questions/comments for consideration:
-In line 94, you state the objective is to assess prevalence among adolescents aged 9-17, but then state in the methods it is just students from 3 grades. In the introduction, I think you could spend a couple more sentences on why the grade 7-9 adolescent population was the population of interest.

**Answer**: Thanks. It is due to the reason that in Afghanistan, 10-12 grade classes are separate and in high school, while <7 grade classes do not have adolescents mostly.

-You state HOW height and weight were measured, but I would add the metrics used (cm, kgs, etc.)

**Answer**: Thanks. OK. Now these have been mentioned clearly.

-In lines 142-145, you discuss the calculation of BMI. I think the article would be strengthened if you added citations that supported the percentile cut offs you used to determine overweight and obese classifications.

**Answer**: Thanks. Now the citations have been mentioned and made clear.

-In the Data Collection section, there was a bit of unnecessary repetition of details already discussed in the Sampling section above it.

**Answer**: Thanks. OK. Now unnecessary repetition of details have been removed from the data collection section.

-Line 251 is incomplete with an error message

**Answer**: Thanks. Now the error message is removed.

-I think there needs to be a little more discussion in how you combined variables to create the variables in Table 6. For example, what made up the categories "does not eat" vs "eats at school"?

**Answer**: Thanks. OK. Now more details have been provided on how we combined the variables to create Table 6.

-I am curious, for the multivariate analysis, did you consider any type of model selection (forward, backward, stepwise)? I am thinking that some of these variables might be masking the effects of others. If you were to use backward selection, for example, and drop TV time, I am curious if the significance of screen time would change? That being said, did you run a correlation analysis to rule out multicollinearity? If you did not do this, I recommend it. If you did do this, add a sentence about it!

**Answer**: Thanks for the great point. Sorry that we didn’t clarify it. Now we have added a sentence in the materials and methods section to clarify it.

-In lines 290-291, you say that the BMI was calculated according to CDC standards. I would add a citation here.

**Answer**: Thanks. Now citation has been added.

-In line 296 you cite a (Rahimi et al., 2025) study. This is not in the references section. How is this paper similar and different to yours? I would discuss it a bit more.

**Answer**: Thanks. Yes, it was a mistake. Now this citation is added in the reference list.

-Are all the studies cited between lines 299-310 on school children that are of similar age to the ones in your study? If not, it may be a stretch to include them. This paragraph should end with discussion of how the prevalence found in your study compares to these other studies - is it lower, higher, about the same?

**Answer**: Thanks. Yes, these references are among school children who were adolescents. The prevalence has now been discussed accordingly.

-In line 360-361, you discuss the use of CDC percentiles, but there has been a lot of literature lately discussing how the CDC percentiles are actually not that acurate for non-white populations. I think it is fine that you are using these percentiles, but am not convinced it is a strength.

**Answer**: Thanks for the point. OK. Now it has been removed from the strenths.
