# Supplementary material for: The prevalence of overweight and obesity and the assessment of associated risk factors among school-aged adolescents in Kandahar City, Afghanistan
Source: PLoS One. 2025 Oct 27;20(10):e0335359. doi: 10.1371/journal.pone.0335359 (PMC12558500; doi:10.1371/journal.pone.0335359)
Supplement: Attachment — Submitted filename: Reviewer Re-Review Report.docx [file pone.0335359.s004.docx]

**Reviewer Re-Review Report**

I have carefully reviewed the revised version of the manuscript titled “The prevalence of overweight and obesity and the assessment of associated risk factors among school-aged adolescents in Kandahar City, Afghanistan.”

The authors have made efforts to revise the manuscript in line with the initial feedback. The previously raised methodological, analytical, and presentation concerns have been adequately addressed. The manuscript now reads more clearly and is better structured overall.

**The authors have:**

a) Clarified the male-only sample limitation and referenced it consistently throughout the Abstract, Methods, Discussion, and Limitations sections.
b) Justified using CDC BMI percentiles in accordance with guidance from the institutional ethics committee.
c) The screen-time threshold and rationale have been clarified based on data distribution, addressing prior confusion about the analytical cutoff.
d) Results tables have been simplified and formatted consistently, and earlier technical errors have been corrected.
e) The Discussion section has been shortened and refocused on key findings, comparisons with regional data, and the study’s novel contributions.
f) The Limitations and Recommendations sections have been strengthened and now directly relate to the findings.

Minor issues, including grammatical inconsistencies, redundant statements, and duplicate references, have been resolved.

While the manuscript has improved, minor editorial polishing by the journal may be needed before publication.

**Recommendation**

The revision has addressed most of the earlier concerns, and the manuscript is now much stronger and clearer. I recommend acceptance after minor editorial or language review, pending confirmation from other reviewers and the handling editor.
